# Pharmaceutical cost and multimorbidity with type 2 diabetes mellitus using electronic health record data

**DOI:** 10.1186/s12913-016-1649-2

**Published:** 2016-08-17

**Authors:** Carla Sancho-Mestre, David Vivas-Consuelo, Luis Alvis-Estrada, Martin Romero, Ruth Usó-Talamantes, Vicent Caballer-Tarazona

**Affiliations:** 1 Research Centre for Health Economics and Management, Universitat Politècnica de València, Edificio 7J, Cno de Vera s/n., 46022 Valencia, Spain; 2 Universidad de Cartagena, Av. del Consulado # Calle 30 No. 48 – 152, Cartagena, Bolívar Colombia; 3 Fundación Salutia, Carrera 71B # 116A-12, PBX: [571] Bogotá D.C, Colombia; 4 Valencian Health Department (Conselleria de Sanitat), General Directorate of Pharmacy and Pharmaceutical Products, Valencia, Spain

**Keywords:** Diabetes mellitus, Pharmaceutical expenditure, Multiborbidity

## Abstract

**Background:**

The objective of the study is to estimate the frequency of multimorbidity in type 2 diabetes patients classified by health statuses in a European region and to determine the impact on pharmaceutical expenditure.

**Methods:**

Cross-sectional study of the inhabitants of a southeastern European region with a population of 5,150,054, using data extracted from Electronic Health Records for 2012. 491,854 diabetic individuals were identified and selected through clinical codes, Clinical Risk Groups and diabetes treatment and/or blood glucose reagent strips. Patients with type 1 diabetes and gestational diabetes were excluded. All measurements were obtained at individual level. The prevalence of common chronic diseases and co-occurrence of diseases was established using factorial analysis.

**Results:**

The estimated prevalence of diabetes was 9.6 %, with nearly 70 % of diabetic patients suffering from more than two comorbidities. The most frequent of these was hypertension, which for the groups of patients in Clinical Risk Groups (CRG) 6 and 7 was 84.3 % and 97.1 % respectively. Regarding age, elderly patients have more probability of suffering complications than younger people. Moreover, women suffer complications more frequently than men, except for retinopathy, which is more common in males. The highest use of insulins, oral antidiabetics (OAD) and combinations was found in diabetic patients who also suffered cardiovascular disease and neoplasms. The average cost for insulin was 153€ and that of OADs 306€. Regarding total pharmaceutical cost, the greatest consumers were patients with comorbidities of respiratory illness and neoplasms, with respective average costs of 2,034.2€ and 1,886.9€.

**Conclusions:**

Diabetes is characterized by the co-occurrence of other diseases, which has implications for disease management and leads to a considerable increase in consumption of medicines for this pathology and, as such, pharmaceutical expenditure.

## Background

Diabetes is regarded as an epidemic by The World Health Organization (WHO) and is one of the non-contagious chronic diseases with greatest socio-economic impact [1]. In Spain, the prevalence of diabetes is estimated to be around 10 % [2]. Worldwide, diabetes-related health expenditure accounted for 11 % of the total health expenditure for 2011 [3].

Diabetes, like the majority of non-contagious chronic diseases, is associated with multimorbitity, defined in the growing literature as the existence of two or more chronic conditions [4, 5]. Multimorbidity causes a negative impact on both clinical and health indicators and primary health care costs [6, 7]. While true that the analysis of multimorbidity in this type of population is relatively new, the tendency towards this approach to the study of chronic diseases is ever increasing [8, 9].

This co-occurrence of diseases has implications from a disease management point of view, as the features of comorbid diseases can be much more complicated than a simple aggregation of individual illnesses [10]. Previous studies have related diabetes to a set of diseases such as cardiovascular, renal, obesity and the metabolic syndrome [11–13].

In this research we use the Clinical Risk Groups (CRG) patient grouping system to identify the diabetic patient and the comorbidity [14–17] CRG is a relatively new system that classifies individuals into mutually exclusive categories, using medical contact or consultation data. Each patient is assigned a severity level according to his/her chronic health conditions. CRGs classify the individual and all the medical services used over a determined period of time. There are 1,079 basic CRGs, all of which can be classified into the CRG core health status that maintain clinical significance and take into account future medical attention needs and the clinical similarities of the individuals assigned to the CRGs. The CRG core health status are: 1) healthy; 2) 4 significant acute disease; 3) single or multiple minor chronic disease; 4) moderate chronic diseases; 5) dominant chronic diseases; 6) Chronic disease in 2 or more organ systems 7) multiple dominant chronic diseases; 8) metastatic malignancies; and 9) catastrophic conditions. These basic groups can be aggregated into three levels (ACRG) with each successive level maintaining the health states and severity, while reducing the number of groups and, if necessary, adjusting the severity level. This grouping system gives a morbidity population pattern.

This study aims to estimate the most common multimorbidities in patients with type 2 diabetes (T2D) and their impact on pharmaceutical expenditure in primary health care using health electronic record data.

## Methods

### Objective

The objective of the study is to estimate the frequency of multimorbidity in type 2 diabetes patients classified by health statuses in a European region and to determine the impact on pharmaceutical expenditure.

### Study population

The study was carried out in the Comunidad Valenciana (CV) in eastern Spain, working with the population for 2012. The total resident population for that year was 5,150,054, from which information was taken on 491,854 patients diagnosed with diabetes, being treated with antidiabetics and/or blood glucose reagent strips. Patients with juvenile diabetes, gestational diabetes and those not receiving pharmacological treatment were excluded, reducing the group to 350,015 patients.

### Data

The study was a cross sectional observational study. The data were obtained retrospectivity from the Electronic Health Record (EHR) and the Electronic Prescriber system (GAIA) corresponding to patients attended during the period January to December of 2012. Each patient has a healthcare identification number in the Population information System (PIS). Cases were identified from the population morbidity database (SCP-cv) of the Directorate General of Pharmacy of the Valencian Community Government Health Department (Conselleria de Sanitat). The population was organized and grouped using CRG. This model makes an initial classification which groups International Classification of Disease, 9^th^ revision, clinical modification (ICD-9-MC) diagnostic codes in Major Diagnostic Categories (MDC). 350,015 people were selected as classified in MDC 101 Diabetes Mellitus (DM) from the ICD-9-MC codes of 250.x0 assigned in their medical encounters in 2012 from a CV total of 5,150,054.

Each MDC group’s different episode disease categories (EDC) also made up from CIE-9-MC codes. It is considered that EDC 427 Diabetes identifies patients with T1D and EDC 424 Diabetes those with T2D. The chronic conditions (EDCs) analyzed in the study as comorbid were: hypertension, dyslipidemia, mental disorders, osteoarticular disease, cardiovascular and cerebrovascular disease (CVD), thyroid disorders, severity obesity, osteoporosis, COPD, cancer, renal disease, Alzheimer, asthma and pulmonary embolism. CVD and Renal failure were included as comorbid still being an event produced by diabetes complications however not being the only cause. For example, a patient diagnosed with diabetes would be in different levels of severity according to comorbidities and complications. A patient with diabetes but without any other associated chronic pathology would be in status 5. Another patient with add on dominant chronic condition, such as hypertension, would be in status 6.

To determine which comorbidities should be considered in this analysis, a review of previous literature was conducted. In addition, we established a consensus with specialists in internal medicine.

Other microvascular diabetes related complications were identified from EHR using codes 205.x2 except for codes 250.10 and 250.12 (indicative of TD2 with ketoacidosis and a condition close associate with T1D) similar to the algorithm proposed by Kho et al. [18]. Nephropathy results are not included here due to them having been insufficiently recorded.

Our algorithm was developed with primarily data warehouse, easily accessed through Structured Query Language (SQL).

### Analysis

From the data obtained, we estimated the frequency of episodes of the chronic diseases in the cohort of patients with T2D selected and classified in CRGs. To study the prevalence of other chronic diseases, we adapt the methodology previously reported by other authors [8]. Following Barnet et al., we display frequencies, percentages and cross tabulations for descriptive analysis. Furthermore, we conduct a binary logistic regression to examine associations between physical and mental health comorbidities. We further add a factorial analysis to study prevalence of common chronic diseases and co-occurrence of diseases and include CRG groups in the descriptive analysis.

An exploratory factor analysis was performed with a varimax rotation applied with the aim of identifying the multimorbidity clusters. Factor analysis is one of the most widely used techniques in building models of multimorbidity [19, 20], although clinical criteria such as grouping by affected organ systems are also pertinent [21, 22].

The EDCs were coded in binary format (i.e., 0 = no disease and 1 = presence of the disease).

The factor analysis was based on a correlation matrix to determine which diagnostic variables comprised each factor [19]. The factors obtained from this analysis were interpreted as multimorbidity patterns. Pharmaceutical use and expenditure were also analyzed bearing in mind the resulting multimorbidity groups. Specifically, we analyzed the use of insulins, oral antidiabetics (OAD) – secretegogues and non-secretegogues – and diabetes test strips, as well as the average cost of these medicines and the total primary health care pharmaceutical expenditure, taking into account the multimorbidity factors obtained.

The incidence of diabetic related microvascular complications (retinopathy, nephropathy and neuropathy) were estimated grouped according to age and gender. Logistic regression identified the effect of gender and age groups (independent variables) on the coexistence of such complications. Pearson chi 2 test was performed to observe the differences between gender and we reported *P* value under 0.05 were considered statistically significant.

The data used was analyzed using the statistics packet SPSS version 20.0. To comply with the data protection law, an anonymous and irreversibly dissociated number replaced the patient’s Healthcare Identification Number (PIS). This observational study was also approved by the Behavioural Research Ethics Board of the *Generalitat Valenciana*.

## Results

### Population description

Taking the total population of people diagnosed with diabetes in the EHR, the estimated gross prevalence in the CV for T2D is 9.6 %, with an average age of 67.4 years. 51.9 % of the population are male (Table 1).

**Table 1:** Number of people with comorbidities, average pharmaceutical expenditure 2012

Variable	Number	Percent
Male	181,658	51.9 %
Female	168,357	48.1 %
Total	350,015	100 %
T2D Comorbidities related		
Hypertension	239,611	68.46 %
Dyslipidemia	186,574	53.30 %
Severe obesity	27,270	7.79 %
T2D Comorbidities Unrelated		
Mental disorders	87,673	25.05 %
Osteoarticular disease	85,920	24.55 %
Thyroid disorders	27,702	7.91 %
Osteoporosis	25,861	7.39 %
COPD	25,561	7.30 %
Cancer	22,792	6.51 %
Alzheimer	14,300	4.09 %
Asthma	11,120	3.18 %
Pulmonary embolism	964	0.28 %
Complications		
Cardiovascular and cerebral disease	50,567	14.45 %
Renal failure	22,496	6.43 %
Retinopathy	25,594	7.31 %
Neuropathy	10,984	3.14 %

Most of patients have diabetes-related comorbidities, mainly hypertension (68 %) and comorbidities not related to diabetes (mental disorders: 25.05 %) (Table 1).

Regarding multimorbidity, 10 % of the diabetic patients suffered from diabetes only, while 70 % suffered from more than 2 comorbidities. The pharmaceutical expenditure shows a clearly rising trend corresponding to the increase in comorbidities (Table 2).

**Table 2:** Number of people with comorbidities, average pharmaceutical expenditure 2012

Number of comorbidities	Total		Average pharmaceutical cost (€)	(standard deviation)
	N	%		
Single	35,644	10.18 %	592.30 €	850.10 €
One additional	73,232	20.92 %	887.80 €	1,008.90 €
Two additional	94,618	27.03 %	1,189.60 €	1,076.60 €
Three additional	72,610	20.74 %	1,531.10 €	1,239.80 €
Four additional	41,505	11.86 %	1,881.60 €	1,371.00 €
Five additional	19,619	5.61 %	2,221.50 €	1,532.90 €
Six and more additional	12787	3.65 %	3,825.41 €	1,802.90 €

Differentiating the population of diabetics according to Core Health Status of CRG, it was observed that as the health of the patient worsens, the prevalence of comorbidities increases, in particular hypertension, dyslipidaemia and cardiovascular disease. Specifically, for Health Statuses CRG 5 to 7 the prevalence of hypertension oscillates between 47.5 % - 97.1 %, while that of dyslipidaemia ranges from 50.9 % to 59 %. For Health Status CRG 8, the prevalence of cancer and hypertension is notable, while for CRG 9 renal disease and hypertension are greater (Table 3).

**Table 3:** Prevalence of comorbidity and events in the diabetic population according to Core Health Status of CRG. 2012

Chronic conditions	CRG 1 to 4 (n)	%	CRG 5 (n)	%	CRG 6 (n)	%	CRG 7 (n)	%	CRG 8 (n)	%	CRG 9 (n)	%				
(EDC)													TOTAL	%	MALE	FEMALE
T2D Comorbidities related																
Hypertension	17,738	38.8	48,659	47.5	140,918	84.3	26,982	97.1	3,728	74.7	1,586	75.4	239,611	68.46 %	117,981	121,630
Dyslipidemia	16,402	35.9	52,082	50.9	97,985	58.7	16,490	59.4	2,473	49.5	1,142	54.3	186,574	53.30 %	96,982	89,592
Severe obesity	2,006	4.4	6,028	5.9	15,647	9.4	3,021	10.9	374	7.5	194	9.2	27,270	7.79 %	11,423	15,847
T2D Comorbidities Unrelated																
Mental disorders	6,208	13.6	18,449	18	49,564	29.7	11,070	39.9	1,742	34.9	640	30.4	87,673	25.05 %	33,274	54,399
Osteoarticular disease	5,610	12.3	18,972	18.5	50,860	30.4	8,957	32.2	1,151	23.1	370	17.6	85,920	24.55 %	29,324	56,596
Thyroid disorders	2,941	6.4	7,245	7.1	14,528	8.7	2,447	8.8	381	7.6	160	7.6	27,702	7.91 %	5,058	22,644
Osteoporosis	1,234	2.7	4,072	4	17,326	10.4	2,655	9.6	356	7.1	218	10.4	25,861	7.39 %	2,427	23,434
COPD	735	1.6	2,386	2.3	10,186	6.1	10,964	39.5	1,010	20.2	280	13.3	25,561	7.30 %	18,307	7,254
Cancer	688	1.5	2,382	23	12,652	7.6	2,159	7.8	4,667	93.5	244	11.6	22,792	6.51 %	13,639	9,153
Alzheimer	447	1	1,661	16	6,556	3.9	5,350	19.3	236	4.7	50	2.4	14,300	4.09 %	4,928	9,372
Asthma	661	1.4	1,590	16	6,932	4.1	1,717	6.2	151	3	69	3.3	11,120	3.18 %	3,381	7,739
Pulmonary embolism	33	0.1	104	1	482	0.3	237	0.9	93	1.9	15	0.7	964	0.28 %	433	531
Complications																
Cardiovascular and cerebral disease	1,463	3.2	7,415	7.2	26,688	16	13,192	47.5	1,215	2.43	594	28.2	50,567	14.45 %	31,135	19,432
Renal disease	1,088	2.4	3,233	32	10,104	6	5,322	19.2	727	146	2,022	96.1	22,496	6.43 %	12,504	9,992

### Multimorbidity characteristics and pharmaceutical expenditure

The factor analysis identifies 6 factors which explain 49.3 % of the variance. The Kaiser-Meyer-Olkin (KMO) statistics value was 0.60 and Bartlett’s test of sphericity was less than 5 %.

The resulting factors were: cardiovascular (hypertension, dyslipidaemia, cardiovascular disease and renal disease), osteoarticular (osteoporosis and osteoarticular disease), mental (mental disorders and Alzheimer), respiratory (asthma and COPD), metabolic (thyroid disorders and obesity) and neoplasms (cancer and pulmonary embolism) (Table 4).

**Table 4:** Factor analysis. Loading with values > ±0.3

	Rotated Component Matrix					
Variables	Component					
	1	2	3	4	5	6
Hypertension	.635					
Dyslipidemia	.596					
Cardiac vascular disease	.540					
Renal disease	.423					
Osteoporosis		.715				
Osteoarticular disease		.639				
Alzheimer			.806			
Mental disorders			.559			
Asthma				.722		
Respiratory illness				.708		
Severe obesity					.676	
Thyroid disorders					.607	
Cancer						.740
Pulmonary embolism						.584

Analyzing pharmaceutical use in diabetic patients according to multimorbidity clusters, uniformity was observed. Diabetic test strips is the most used technology in diabetic patients. Secretagogue OADs are the most used in patients with cardiovascular, osteoarticular and metabolic factors (Table 5).

**Table 5:** Frequency of medicine use and average pharmaceutical cost by multimorbidity groups

Medicine	Factor 1 Cardiovascular		Factor 2 Osteoarticular (%)	Factor 3 Mental (%)	Factor 4 Respiratory (%)	Factor 5 Metabolic	Factor 6 Neoplasm
						(%)	(%)
Insulins	N (%)	6.8 %	6.5 %	7.8 %	8.2 %	7.5 %	8.3 %
Insulins	Pharmaceutical expenditure (€)	122.4	118.1	133.5	139.9	146.6	125.6
	(standard deviation)	(307.1)	(298.7)	(314.3)	(330.2)	(361.5)	(296.7)
Secretagogue OADs	N (%)	32.9 %	32.5 %	31.7 %	30.5 %	32.8 %	30.9 %
Secretagogues	Pharmaceutical expenditure (€)	16.0	16.4	15.3	14.5	14.7	15.2
	(standard deviation)	(24.0)	(24.6)	(23.2)	(22.9)	(22.0)	(23.9)
Nonsecretagogue OADs	N (%)	10.9 %	10.6 %	10.3 %	10.4 %	10.5 %	10.2 %
Non	Pharmaceutical expenditure (€)	226.2	213.5	206.3	202.0	239.6	191.1
secretagogues	(standard deviation)	(362.6)	(352.4)	(349.3)	(342.3)	(415.3)	(328.8)
Strips	N (%)	31.5 %	31.8 %	33.9 %	31.7 %	32.5 %	37.0 %
Strips	Pharmaceutical expenditure (€)	222.8	227.8	235.6	241.4	246.3	225.1
	(standard deviation)	(192.7)	(192.8)	(200.2)	(203.4)	(217.0)	(191.5)
Average total cost	Pharmaceutical expenditure (€)	1,441.0	1,645.5	1,816.7	2,123.1	1,540.6	1,968.9
	(standard deviation)	(1,290.4)	(1,357.0)	(1,570.6)	(1,590.1)	(1,421.9)	(1,685.8)

The highest average expenditure on insulin is for those belonging to the metabolic factor, equivalent to 146.6 €. The highest average expenditure on secretagogue OADs corresponds to the osteoarticular factor, at 16.4€. For nonsecretagogue OADs the highest expenditure was also on patients in the metabolic factor, at 239.6 €. Patients in the metabolic factor also had the highest average cost in diabetic strips at 246.3 €. The group of patients in the respiratory factor showed the highest average total pharmaceutical expenditure of 2123.1€ (Table 5).

### Analysis of diabetes microvascular complications

The prevalence of patients with T2D with microvascular complications with retinopathy (7.31 %), and neuropathy (3.64 %). Renal failure (6.43 %) and patients with cardiovascular and cerebrovascular disease (14.45 %).

In the logistic regression the microvascular complications are related to the age and gender of the patients. All the variables were significant. The reference is the man of less than 40 years of age. From the logistic regression we obtain some conclusions: regarding to gender, women present lower probability than men of suffering retinopathy, and more probability of suffering the rest of the complications. Regarding age; older patients, presents a higher probability of suffering all of the complications mentioned (Table 6).

**Table 6:** Logistic regressions of diabetes-related complications adjusted according to age and gender

	Diabetic Nephropathy		Diabetic Neuropathy		Retinopathy	
	OR	*P* value	OR	*P* value	OR	*P* value
Gender						
Females	0.23	0.00	0.17	0.00	−0.09	0.00
Age bands						
Range 40–44	0.32	0.00	0.55	0.00	0.33	0.00
Range 44–49	0.42	0.00	0.87	0.00	0.52	0.00
Range 50–54	0.39	0.00	1.00	0.00	0.59	0.00
Range 55–59	0.54	0.00	1.05	0.00	0.69	0.00
Range 60–64	0.57	0.00	1.16	0.00	0.80	0.00
Range 65–69	0.76	0.00	1.21	0.00	0.91	0.00
Range 70–74	0.91	0.00	1.27	0.00	0.91	0.00
Range 74–79	1.01	0.00	1.47	0.00	0.90	0.00
Range 80–84	1.18	0.00	1.47	0.00	0.80	0.00
Range 85 and over	1.17	0.00	1.38	0.00	0.61	0.00
Reference (Male <40)	−4.22	0.00	−4.75	0.00	−3.27	0.00

*OR* Odds ratio. Statistically significant subgroups are those with *P* value <0.05

## Discussion

In this study, both the prevalence of diabetes and the comorbidities are based on information from the EHR and not from other sources usually used in cross sectional studies. The use of the EHR for automated and subsequent analysis of patients with DM2 is not novel, and a base of evidence is beginning to be built on this. Some authors have published similar algorithms to our system that allow automatic identification of diabetic patients.

Klompas et al. showed that using EHR data for automated diabetes surveillance was feasible and of great potential utility. By taking advantage of the complete data offered by EHRs, including laboratory test results and prescriptions as well as diagnosis codes, an increase can be achieved in both the sensitivity and detail of the surveillance over the use of diagnosis codes alone. Using an integrated algorithm, including laboratory, prescription, and ICD-9 criteria, more patients with diabetes are captured than when using any one criterion alone [23].

Regarding to the prevalence of patients who Suffered DM microvascular complications: first, retinopathy (7.31 %), whose prevalence is very similar to that of other authors (7.8 %) but far from the Pantalone et al. (3.4 %) [24]. The second complication was the neuropathy (3.64 %) with a prevalence above the indicated by Alonso Moran et al. [25] (1.3 %) and below that of Pantalone (18 %). Finally, the prevalence of nephropathy was also a lower figure (3.14 %) to that of the Alonso Moran et al. (8 %) [26].

The main contribution of this study is the relation between diabetes co-morbidity and pharmaceutical expenditure. The relationship with the CRG patient classification system, a clear trend in the increase of pharmaceutical consumption was observed with the more serious health states. In the case of the analyzed population, multimorbidity was present in nearly all cases, which is consistent with the results of other studies [3, 16, 24, 27, 28] and similar to that of Giralt [29].

Regarding the demographic profile of the population (gender and age) findings were very similar to previous studies [25]. In comparison with other studies, as the age increases the likelihood of suffering complications also increases [30].

The highest pharmaceutical cost implications are connected to mental factors, being higher than that of other factors such as cardiovascular, metabolic and the osteoarticular. This is consistent with recent evidence indicating that the presence of one or more mental comorbidities in people with diabetes may increase the cost from 15 to 94 % [31].

The results were similar to those reported by Huber, which is US $ 857 for diabetes alone, US $ 1,298 for the presence of comorbidity, and US $ 1,795 for the presence of two comorbidities. The percentage of people with comorbidities increases, two of those percentages being similar to those reported by Huber [32].

As reported by Huber, the higher cost factor is of respiratory. However, the cost found in this study is higher than that estimated by Huber.

The main limitation regarding the estimates with using this information system is the dependency placed on the accurate recording of the patients’ data in the EHR by the health professionals involved. Thus, the percentage of DM remains more or less constant and requires improvement in the recording of the codes.

## Conclusions

Diabetes is characterized by the co-occurrence of other diseases, which has implications from a disease management point of view, and means a considerable increase in pharmaceutical consumption for this pathology and, as such, for pharmaceutical expenditure.

Additional studies are necessary for better characterization of the development of the disease and multimorbidity throughout life to establish intervention priorities.

## Abbreviations

CRG: Clinical Risk Groups
CV: Comunidad Valenciana
CVD: Cerebrovascular Disease
DM: Diabetes Mellitus
EDC: Episode Disease Categories
EHR: Electronic Health Record
ICD-9-MC: International Classification of Disease, 9^th^ Revision, Clinical Modification
KMO: Kaiser-Meyer-Olkin
MDC: Major Diagnostic Categories
OAD: Oral Antidiabetics
PIS: Population Information System
SQL: Structured Query Language
T2D: Type 2 Diabetes
WHO: World Health Organization
